# Strain Assessment of Deep Fascia of the Thigh During Leg Movement: An *in situ* Study

**DOI:** 10.3389/fbioe.2020.00750

**Published:** 2020-07-29

**Authors:** Yuliia Sednieva, Anthony Viste, Alexandre Naaim, Karine Bruyère-Garnier, Laure-Lise Gras

**Affiliations:** ^1^Univ Lyon, Université Claude Bernard Lyon 1, Univ Gustave Eiffel, IFSTTAR, LBMC UMR_T9406, Lyon, France; ^2^Hospices Civils de Lyon, Hôpital Lyon Sud, Chirurgie Orthopédique, Pierre-Bénite, France

**Keywords:** deep fascia of the thigh, digital image correlation, motion analysis, strain, *in situ* study, iliotibial tract, biomechanics

## Abstract

Fascia is a fibrous connective tissue present all over the body. At the lower limb level, the deep fascia that is overlying muscles of the outer thigh and sheathing them (fascia lata) is involved in various pathologies. However, the understanding and quantification of the mechanisms involved in these sheathing effects are still unclear. The aim of this study is to observe and quantify the strain field of the fascia lata, including the iliotibial tract (ITT), during a passive movement of the knee. Three fresh postmortem human subjects were studied. To measure hip and knee angles during knee flexion-extension, passive movements from 0° to around 120° were recorded with a motion analysis system and strain fields of the fascia were acquired using digital image correlation. Strains were computed for three areas of the fascia lata: anterior fascia, lateral fascia, and ITT. Mean principal strains showed different strain mechanisms depending on location on the fascia and knee angle. For the ITT, two strain mechanisms were observed depending on knee movement: compression is observed when the knee is extended relative to the reference position of 47°, however, tension and pure shear can be observed when the knee is flexed. For the anterior and lateral fascia, in most cases, minor strain is higher than major strain in absolute value, suggesting high tissue compression probably due to microstructural fiber rearrangements. This *in situ* study is the first attempt to quantify the superficial strain field of fascia lata during passive leg movement. The study presents some limitations but provides a step in understanding strain mechanism of the fascia lata during passive knee movement.

## Introduction

Pathologies of the musculoskeletal system have become a major public health issue, especially due to an aging and sedentary population. Osteoarticular pathologies such as osteoporosis or musculoskeletal disorders lead to disability and loss of autonomy. In the lower limb, for instance, several pathologies such as osteoarthritis, iliotibial band syndrome, inflammatory lesions, joint disorders, traumatic injuries etc. may significantly affect a patient’s quality of life. To develop a greater understanding of these pathologies and to provide better health care, clinicians are increasingly using biomechanical modeling (Chèze et al., 2012). Musculoskeletal models allow the study of everyday life movements – walking, climbing stairs, getting up from a chair – thanks to kinematic and dynamic analyses, and can therefore provide a thorough evaluation of patients’ functional status.

Dynamic multi rigid body models of the lower limb (Moissenet et al., 2014) can predict musculotendinous forces within the joints as well as ligament and contact forces. However, the main limitation of these models is that muscles are represented only by their main lines of action. They are unable to reproduce forces induced transversely (Huijing, 2009) by a muscle’s activation on other muscles, bones and joints via fascias, epimysium and connective tissues.

To overcome this limitation, finite element models of the lower extremity including contractile muscular volumes (Stelletta et al., 2016, 2017) or for one specific muscle (Westman et al., 2019) have been developed. Nevertheless, even though these models include detailed geometry and mechanical properties of muscles, improvements are still needed to increase their biofidelity in reproducing the complex passive mechanical action of soft connective tissues, especially the fascias. These passive structures sheath muscles and muscular compartments; exert a stress or pressure on them and thus participate in force transmission in both longitudinal and transverse directions during muscle activation (Huijing, 2009; Snoeck et al., 2014; Eng and Roberts, 2018). With their finite element model of the upper leg where contacts between muscles were defined by contact laws, Stelletta et al. (2016, 2017) showed that in order to properly reproduce muscle activation in three dimensions, these connections were not enough to replicate transverse loadings. They therefore suggest that including connective tissues and fascias in the model would improve its response. To do so, mechanical behavior, and strain levels of fascias for both passive and active movements are required to be implemented in such models.

Fascias can be either loose or dense connective tissues. Deep fascias are multi-layered dense fibrous connective tissues, mainly made of collagen and elastin fibers (Stecco et al., 2011), and surround muscles and groups of muscles. It has been shown that they contribute to the pressure in the muscular compartments (Garfin et al., 1981), and to muscle force transmission (Snoeck et al., 2014), they transmit mechanical forces between muscles (Huijing, 2009); they also participate in movement coordination (Barker et al., 2004), limb stabilization (Stahl, 2010), and elastic energy storage (Bennett et al., 1986).

Many studies have been carried out to characterize the mechanical behavior of deep fascias in relationship with their microstructure. These tissues are anisotropic, non-linear viscoelastic materials (Findley et al., 2015). *In vitro* tests and associated constitutive models have been proposed to characterize these tissues (Eng et al., 2014; Ruiz-Alejos et al., 2016; Otsuka et al., 2018). Presented results are obtained from animal tissue (Eng et al., 2014; Ruiz-Alejos et al., 2016) or human tissue (Stecco et al., 2014; Otsuka et al., 2018). They were obtained from unidirectional (Pavan et al., 2015; Ruiz-Alejos et al., 2016; Otsuka et al., 2018) or biaxial (Eng et al., 2014; Pancheri et al., 2014) tensile tests performed until rupture or at high strain levels (Stecco et al., 2014; Ruiz-Alejos et al., 2016). Pure shear loading (Ruiz-Alejos et al., 2016) was also studied and used to identify a constitutive law.

Biaxial testing aims at reproducing a more realistic loading of deep fascias than uniaxial tension, and tests to failure help to understand the mechanical behavior of the deep fascias under large strains. However, the mechanical behavior (mechanism, level of strain) of the deep fascias during movement is relatively unknown.

In this study, we focus on the fascia lata, which overlies the muscles of the outer thigh. It is involved in various pathologies, such as iliotibial band syndrome (Fairclough et al., 2006; Huang et al., 2013), compartment syndrome (Nau et al., 2000), varus-valgus of the knee (Clarke et al., 2005; Nikolopoulos et al., 2015), and traumatic injuries. As part of the fascia lata, the iliotibial tract (ITT) shows complex interactions with muscles and skeletal structure of the lower limb, and has a major role in the knee joint stability (Fairclough et al., 2006; Vieira et al., 2007).

The aim of this study is to observe and quantify the strain field of the external surface of the fascia lata, including the ITT, during a passive movement of knee flexion-extension. Collected data could then be useful to perform adequate sample testing and develop constitutive laws of fascia to be implemented in numerical models dedicated to clinical applications. This analysis is based on *in situ* testing and involves movement analysis and strain field measurement using stereovision and digital image correlation.

## Materials and Methods

### Specimen Preparation

Three fresh postmortem human subjects from the Department of Anatomy of the University of Rockefeller (DAUR), Lyon, France, were instrumented for motion analysis of the left leg and the measurement of the strain field on fascia lata during knee flexion-extension movement. Ethical approval was not required for this study since it is not required by French law. The cadaver subjects were obtained after the deceased gave their informed consent to donate them to science during their lifetime (law article R2213-13). The authors ensure that these experiments were performed with respect for these remains, and in correct health and safety conditions. These subjects were in average 92.3 years-old (±2.5), measured 161 cm (±2.6 cm), weighted 45.5 kg (±12.3 kg) and had a BMI of 13.6, 16.6, and 22.3 kg/m^2^, respectively. Subjects were considered as fresh since no specific body preservation was performed: no injection of any embalming solution, no freezing. After death, bodies were kept at 4°C until testing. The duration between time of death and time of test was 6 days (5 days postmortem and 1 additional day for body preparation) for subject 2019_285, it was 5 days (4 + 1 days postmortem) for subject 2019_300 and 3 days (2 + 1 days postmortem) for subject 2019_308. The average postmortem interval was 4.7 days.

**FIGURE 1:**
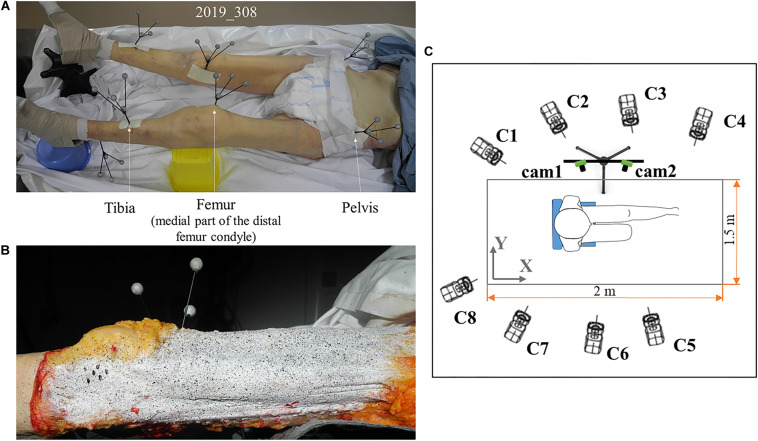
Subject preparation and testing area: **(A)** tripods of markers for optical motion capture are screwed to the tibia, femur, and pelvis. These tripods allow for the analysis of leg movement and knee angles. To avoid contact between tripods of the right leg and those of the left leg, the left knee is elevated supported by the yellow box underneath it; **(B)** speckle pattern on fascia lata for stereo digital image correlation. White mask is applied to standardize the background and for good contrast of the speckle pattern created with black ink. **(C)** Motion capture and digital image correlation camera positions. Eight cameras (C1 to C8) for motion capture are placed around the chair where the subject is positioned. Camera positions are optimized so each tripod of markers can be seen by at least three cameras. Acquisition volume is 2 × 1.5 × 2 m^3^. Two cameras (cam1, cam2) for digital image correlation are placed perpendicular to the left leg of the subject and focused on it to acquire images of the speckle on lateral fascia and ITT during leg movement. These cameras can be moved to focus on the top of the leg to acquire strain field on anterior fascia.

For motion analysis, six rigid tripods with attached markers (four reflective markers of 10 mm diameter fixed to a rod) were screwed in the right and left areas of the pelvis, in both femurs, and in both tibias (Figure 1A). Particular attention was paid to the position of the tripods in the femur. They were implanted in the medial condyles in order to avoid any interference with the lateral and anterior areas of the thigh where soft tissue strains were measured.

In order to measure the superficial strain of the deep fascia lata by using the digital image correlation, a random speckle pattern was applied to the surface of the fascia lata after skin and fat removal (Figure 1B). To do this, the fascia lata was covered with a thin layer of white clown makeup to standardize the background and obtain good contrast, speckles were then created using a toothbrush and black ink to apply droplets on the white surface (Podwojewski et al., 2014, 2013). The washable white makeup layer could be easily removed without damaging the tissue, and therefore it was possible to redo the pattern until an appropriate speckle size or density for digital image correlation was obtained. Initially, speckles were created on the anterior area of the fascia lata, and after testing with observation of this area, speckles were created on the lateral side of the fascia for the new testing. This procedure allowed for correct tissue hydration, keeping it in saline solution when not being tested. It also reduced the risk of erasing the speckle during testing.

During tests, the body was placed on a specific chair at ambient temperature. This chair facilitated the installation of the subject lying on its back with buttocks at the edge of the seat, and then back rest support reproducing a seated posture, both legs were suspended allowing full knee flexion.

### Anatomical Frames Acquisition and Motion Analysis

The acquisition was performed using an optical motion capture system – Optitrack – with 8 cameras (1280 × 1024) recording at 100 Hz. The 8 cameras were oriented in order to cover a volume of interest (2 × 1.5 × 2 m^3^) where each tripod could be seen by at least three cameras at the same time during the leg movement from extension to flexion (Figure 1C). Considering the volume of interest (Eichelberger et al., 2016) and the optical motion capture system used (Carse et al., 2013), eight cameras were sufficient to accurately measure leg kinematics. Each tripod screwed into the lower limbs meant that a technical frame for each body segment could be defined, to follow its position during movement. In order to define the anatomical frame relative to these technical frames, anatomical markers were palpated with an additional tripod when the body was lying on its back. Each tripod consisted of four markers fixed to a rod.

The tip position of the additional tripod used for palpation was previously calibrated. Calibration was performed by pointing three points of known positions. As in the lying position the posterior superior iliac spine was inaccessible, it was supposed that the mid sacrum marker was positioned vertically relative to the middle of the left and right anterior superior iliac spine on the seat plane. All anatomical points are listed in Table 1. Joint kinematics were then calculated using the anatomical frame and Euler sequences recommended by the International Society of Biomechanics (Wu et al., 2002) using MATLAB. During acquisition, hip angle varied: for subject 2019_285 it was 24.84 ± 10.75°, for subject 2019_300 it was 26.71 ± 9.45° and for subject 2019_308 it was 20.38 ± 8.63°.

**TABLE 1:** List of anatomical points and associated anatomical frames.

**Anatomical points**
Left and right anterior superior iliac spine
Left and right greater trochanter
Left and right lateral epicondyle
Left and right medial epicondyle
Left and right fibula head
Left and right tibial tuberosity
Left and right lateral malleolus
Left and right medial malleolus

### Digital Image Correlation

In order to obtain the 3D surface strain field of the thigh fascia, a digital image correlation set-up was required. A pair of low noise cameras were rigidly mounted and fixed in place during system calibration and subsequent data acquisition (Figure 1C). The cameras used for these tests were GO-5000-USB from JAI’s Go Series (maximum frame rate: 62 Hz) with objective KOWA LM12HC with lenses of 12.5 mm and an f-stop range of f/14 to f/16. Image size was 2560 × 2048 px. The frame rate for the acquisition was set to 50 Hz, two times lower than for the motion analysis recording. This chosen frame rate allowed an easier synchronization and correspondence between strain measurements and knee angles, while it resulted in at least 300 data points for each flexion or extension knee movement. The depth of field was optimized by closing the diaphragms as much as possible: an aperture of f/16 was kept consistent during and between acquisitions, permitting a correct focus on the area of interest although slight changes in the distance between the area of interest and the cameras. The light was adjusted to optimize image quality and for a sharp contrast. Owing to this configuration, strains in planes closer or further from the cameras’ focal plane were captured thanks to clear high-quality images. Care was taken to match cameras center points. Calibration was carried out using a calibration grid adapted to the size of the field of view with a 5 Hz frame rate.

Data processing was carried out with VIC-3D software. For the area of the ITT and lateral fascia, the size of pixels was 0.19 mm; for the anterior fascia, the size of pixels was 0.18 mm. The subset was chosen equal to 31 and the step at 7.

For this strain analysis, a hip angle of 26.52 ± 3.85° (respectively 25.23°, 30.41°, and 23.91° for each subject) and knee flexion of 47 ± 0.5° was considered as a reference image for strain analysis. This angle for the knee was chosen due to the position of the body with zero gravity (Mount et al., 2003).

**FIGURE 2:**
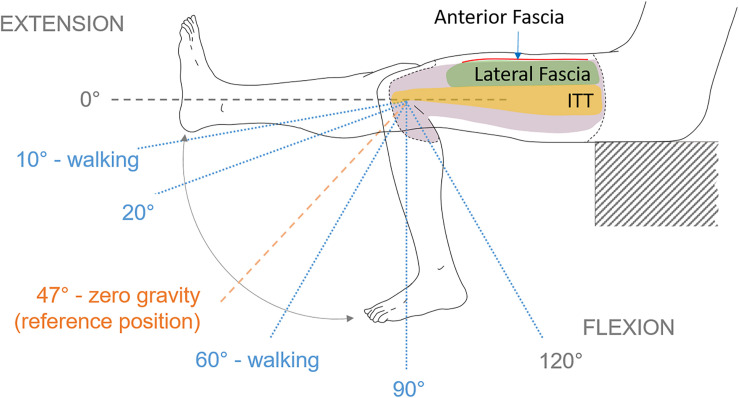
Lateral view of the thigh during the leg flexion-extension movement with angles and areas of interest identification. Lateral fascia, anterior fascia and ITT are the three areas of interest. The leg movement starts from full extension of the knee until maximum reachable flexion. Knee angle of 47°corresponds to the reference position used for digital image correlation. Angles of 20°, 60°, and 90° are arbitrary chosen to look at strain fields.

For the lateral view, the area of interest was divided into “ITT area” and “above ITT area” named “lateral fascia” (Figure 2). The anterior view area of the fascia was entirely post processed and named “anterior fascia.” Major principal (E_1_), minor principal (E_2_) and maximum shear (maxE_xy_) Green-Lagrange strains were extracted.

### Test Conductions

The knee extension-flexion range of motion was applied to the left leg manually from 0° (full extension) to 120° (flexion) (Figure 2). These movements were chosen in order to cover the possible positions of the leg during daily life activities (walking, step climbing, etc.) and stretching.

During the leg movement, care was taken to avoid subject contact with the experimental setup, keeping the femur parallel to the digital image correlation system and as motionless as possible to stay in the field of view, and to maintain focus on the surface of interest. Movements were recorded with the anterior area of the fascia lata as region of interest for the digital image correlation. The digital image correlation system was then moved to record strains on the lateral side of the fascia. For each area of interest, three tests consisting of five cycles of knee flexion-extension were performed. Cycles were applied continuously. A waiting time of 10 min was observed between tests.

Motion analysis and digital image correlation acquisitions were synchronized using a common trigger box sending a rising edge square signal to both systems. All the data extracted from motion analysis and digital image correlation were processed using a custom-made script in Python. Due to the stretching of knee area during knee flexion and creation of folds during the full extension, strain could not be computed on a sufficient number of points in this area. Therefore, the knee area was excluded from fascia strain computations.

## Results

### Knee Angular Velocity

**FIGURE 3:**
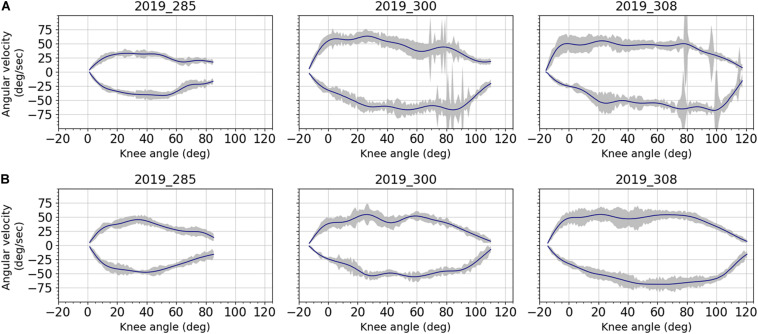
Averaged angular velocities and corresponding standard deviations (gray area) over the knee angles for all subjects: **(A)** anterior fascia; **(B)** lateral fascia and ITT. Data were filtered using a forward-backward linear filter. Filter coefficients were obtained from a low-pass digital Butterworth filter (3rd order, cut off frequency of 2Hz) that was first applied on data. Even though angular velocity was applied manually, results show good reproducibility for each subject. Inter-subject variability can be observed because of subjects’ different knee mobility and associated range of motion.

Figure 3 illustrates the knee angular velocity recorded during movement for each subject and for each digital image correlation configuration: observing the anterior fascia and observing both the lateral fascia and ITT. Although leg movement was applied manually, angular velocity was relatively reproducible throughout the five cycles of each configuration and over the three tests for each subject. Relative standard deviations (RSD) ranged from 21 to 28% with a mean value of 24% for tests on anterior fascia. For tests performed on lateral fascia and ITT, they ranged from 18 to 25% with a mean value of 22.5%. Maximum angular velocities in flexion (positive values) and in extension (negative values) were close in absolute value, with an average difference of 7 deg/sec considering the three subjects and two configurations. For each subject, the angular velocities were also similar between the two configurations, with an average difference in maximum velocity during flexion of 8 deg/sec.

### Strain Analysis

#### Average Strains

**FIGURE 4:**
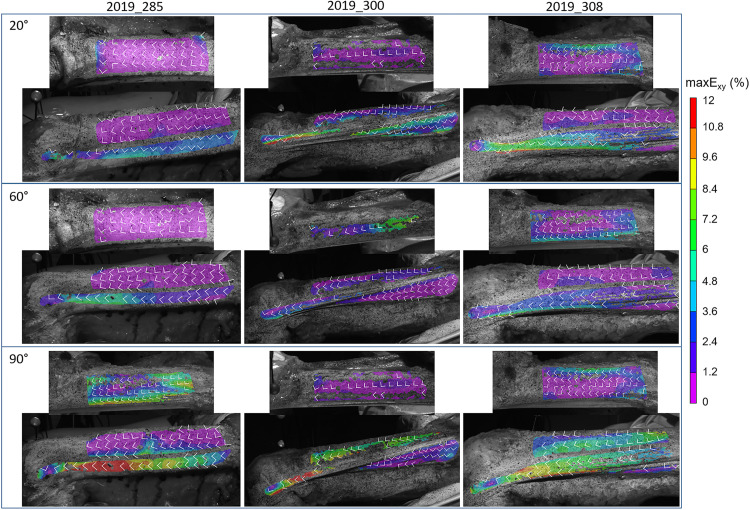
Maximal shear strain distribution maxE_xy_ on the anterior fascia, lateral fascia, and ITT, at knee angles of 20 ± 0.5°, 60 ± 0.5°, and 90 ± 0.5° for all subjects (Test 2). White arrows show the direction of the major E_1_ and minor E_2_ principal strains, which are perpendicular to each other. For instance, for the first line knee angle is 20°, the top images are anterior fascia, while lower images show lateral fascia and ITT.

Shear strain fields obtained for three knee angles are given for all areas of fascia and ITT and for each subject and for three knee angles (20°, 60°, and 90°), as shown in Figure 4.

**FIGURE 5:**
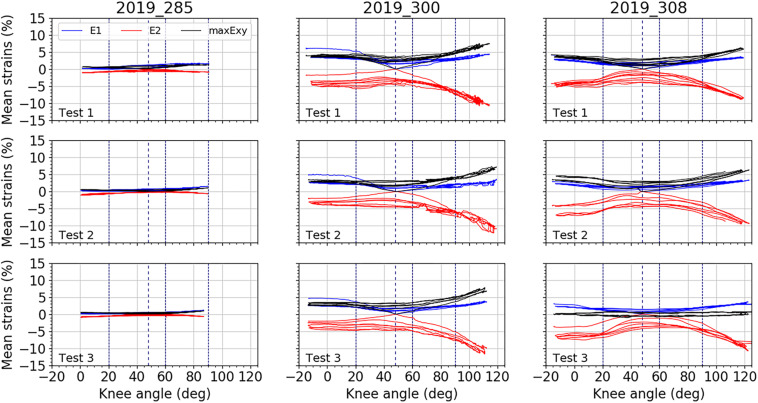
Mean strain distributions over the knee angles for the anterior fascia. In blue, mean major principal strain E_1_, in red, minor principal strain E_2_, and in black, maximal shear strain maxE_xy_. For each test, all cycles are plotted. Vertical dashed line represents the knee position chosen as reference: 47°. Three other vertical lines locate knee angles of 20°, 60°, and 90°.

**FIGURE 6:**
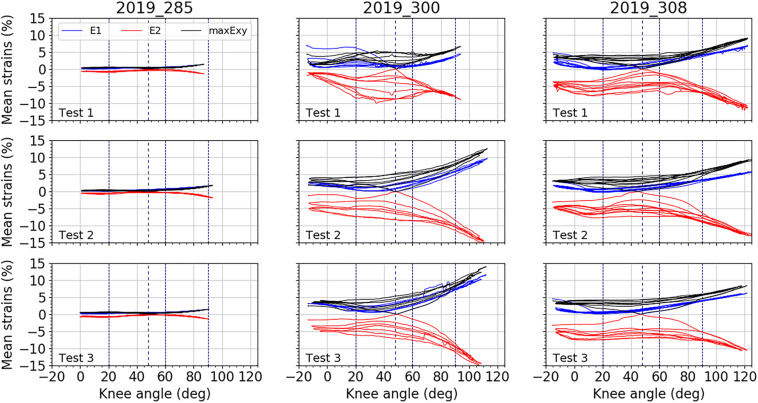
Mean strain distributions over the knee angles for the lateral fascia. In blue, mean major principal strain E_1_, in red, minor principal strain E_2_, and in black maximal shear strain maxE_xy_. For each test, all cycles are plotted. Vertical dashed line represents the knee position chosen as reference: 47°. Three other vertical lines locate knee angles of 20°, 60°, and 90°.

**FIGURE 7:**
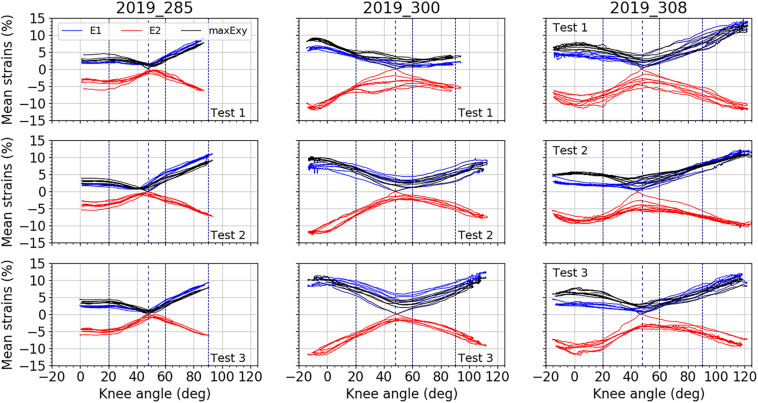
Mean strain distributions over the knee angles for the ITT. In blue, mean major principal strain E_1_, in red, minor principal strain E_2_, and in black maximal shear strain maxE_xy_. For each test, all cycles are plotted. Vertical dashed line represents the knee position chosen as reference: 47°. Three other vertical lines locate knee angles of 20°, 60°, and 90°.

Figures 5–7 illustrate the average strains computed along the five cycles of the three tests performed on each subject, on the area of the anterior fascia, the lateral fascia and the ITT, respectively. For each subject, we can observe a good reproducibility of results over the five cycles and the three tests, except on the lateral area of the fascia for which we can see a larger dispersion of the minor strain levels along the cycles. As for RSD, each area of the fascia for both major and minor strains are presented in Table 2. For tests on the anterior fascia, RSD were in average ranging from 20 to 53%. Similar results were obtained for the lateral fascia, with RSD ranging from 20 to 39%. Results were less dispersed for the ITT with RSD ranging in average from 15 to 25%. For all curves, maximum RSD was obtained for knee angles around the reference position of 47°, which can be seen on Figures 5–7.

**TABLE 2:** Mean, standard deviation (SD) and maximum relative standard deviations (RSD) for each area of fascia, for each subject, and for both major E_1_ and minor E_2_ strains.

Fascia area	Subject #	mean RSD for E_1_ (%)	SD RSD for E_1_ (%)	max RSD for E_1_ (%)	mean RSD for E_2_ (%)	SD RSD for E_2_ (%)	max RSD for E_2_ (%)
Anterior	2019_285	43	26	81	53	71	100
	2019_300	22	13	60	21	12	48
	2019_308	20	13	55	21	13	62
	***mean***	***28.3***			***31.7***		
Lateral	2019_285	29	15	54	26	16	71
	2019_300	31	20	71	25	15	57
	2019_308	39	33	71	20	13	57
	***mean***	***33***			***23.7***		
ITT	2019_285	15	9	43	19	12	54
	2019_300	21	13	56	25	20	85
	2019_308	20	14	77	16	11	55
	***mean***	***18.7***			***20***		

*RSD were calculated considering the five cycles and the three performed tests. Experimental results were overall less dispersed for ITT area. Italicized values are mean values for the three subjects.*

For the subject 2019_285, on both the lateral and anterior areas of the fascia, we measured lower strains compared to the other subjects. For a knee angle of 90° (flexion), the average shear strain values on the fascia did not exceed 2%. Strains of the ITT are also lower in full extension of the knee. The specific behavior of this subject was associated to a subjective observation of knee rigidity in rotation and varus-valgus during the experiment compared to the other subjects. Moreover, full extension of the knee was limited for this subject compared to the others with a minimum knee angle of 0° while it was around −15° for other subjects.

**FIGURE 8:**
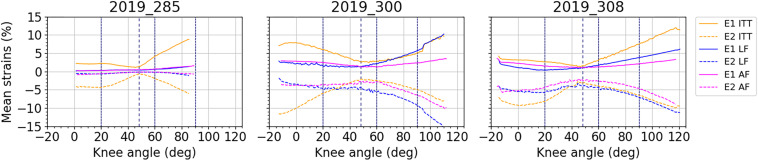
Average curves for mean major principal strain E_1_ and mean minor principal strain E_2_ over the knee angles and by area of interest: ITT: yellow curves; lateral fascia (LF): blue curves; anterior fascia (AF): magenta curves.

For each subject, average curves obtained for the different areas of fascia and ITT are illustrated in Figure 8. A large inter-subject variability of fascia and ITT behavior is observed. This can be related to the variability of anthropometries of the three subjects, specifically in terms of BMI or to other physical conditions that could affect soft tissue mechanical behavior.

#### Strain Mechanisms

**FIGURE 9:**
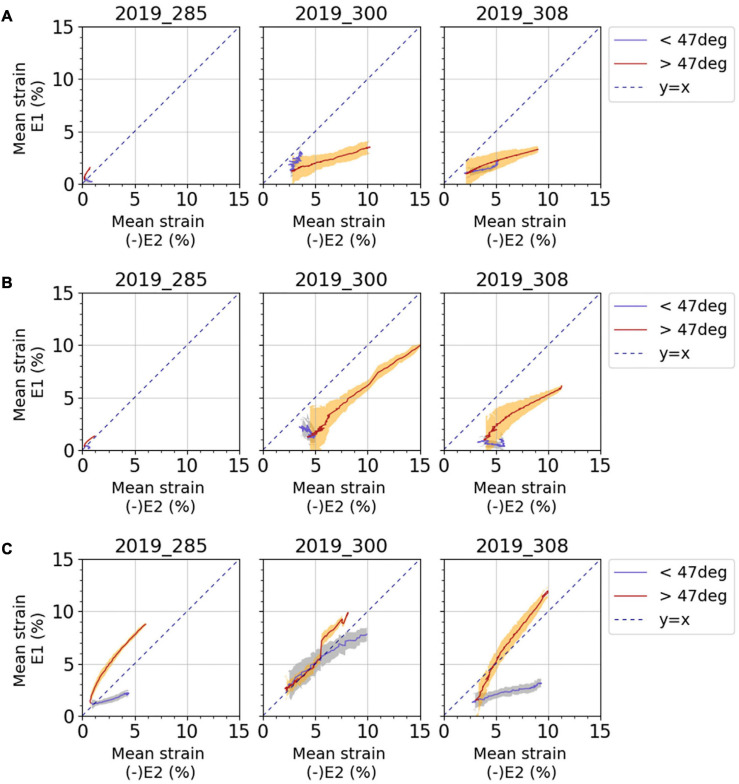
Average curves for mean major principal strain E_1_ against negative mean minor principal strain E_2_ for **(A)** anterior fascia, **(B)** lateral fascia, and **(C)** ITT. Results are presented for each subject and for each test. Blue curves and light blue areas correspond respectively to average values and standard deviation obtained for a knee angle lower than the reference position of 47°: extended knee. Red curves and orange areas correspond respectively to average values and standard deviation obtained for a knee angle greater than the reference position of 47°: flexed knee. Dashed line represents the y = x curve; when points are along this line, pure shear can be observed.

Despite the apparent inter-subject variability, the evolutions of major and minor strains along the knee flexion-extension movement seem vertically mirrored in many cases. To analyze the E_1_/E_2_ ratio, Figure 9 illustrates E_1_ against (−E_2_). On this figure, two phases of the movement corresponding to postures closer to knee flexion (i.e., knee angles > the reference knee angle of 47°) and the postures closer to knee extension (i.e., knee angles < the reference knee angle of 47°), are illustrated with two different colors.

On the anterior fascia (Figure 9A), for subject 2019_285, for which strain levels are lower, a tension mechanism is observed with a maximum average tensile strain around 1.5% and a lower absolute value of compressive strain around 0.5%. For subjects 2019_300 and 2019_308, absolute value of the compressive strain is greater than the average tensile strain when the knee is more flexed relative to the reference knee angle (red curves, knee angle > reference angle).

On the lateral fascia (Figure 9B), for the subjects 2019_300 and 2019_308, the same analysis as for the anterior fascia can be made, and compressive strain can reach up to 15% in absolute value. For the subject 2019_285, for which strain levels are lower, the strain mechanism of the fascia is pure shear with a ratio E_1_/(−E_2_) equal to 1 when the knee is more flexed relative to the reference knee angle (red curves, knee angle > reference angle).

On the ITT (Figure 9C), the two phases of knee movement show different strain mechanisms for subjects 2019_285 and 2019_308. When the knee is more flexed relative to the reference knee angle (red curves, knee angle > reference angle), even if a tensile strain larger than the compressive strain may be observed, we can observe pure shear with a curve gradient close to 1. For subject 2019_300, the two phases of movement do not differentiate between strain mechanisms so clearly, but we can also observe some pure shear. Considering all tests, strain measurements on ITT show maximum tensile strain between 2 and 15% and compressive strain between 4 and 13% in absolute values.

### Strain Rates

**FIGURE 10:**
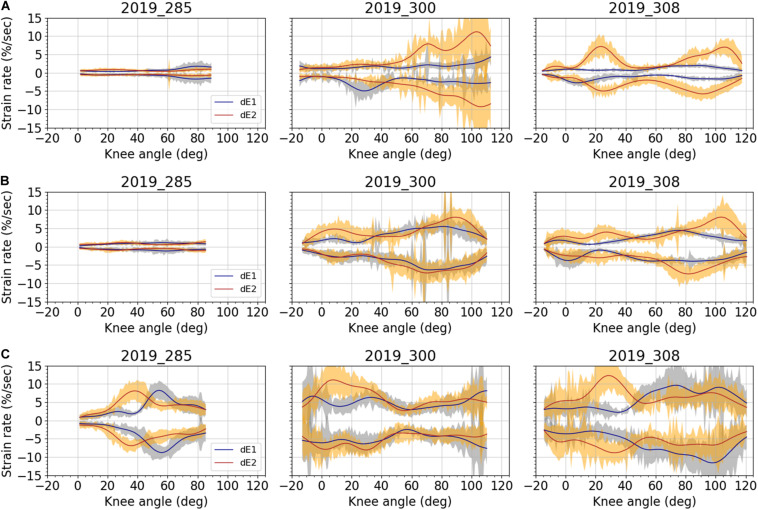
Averaged strain rates and corresponding standard deviation over the knee angles for all subjects: **(A)** anterior fascia; **(B)** lateral fascia; and **(C)** ITT. Blue curves and light blue areas correspond respectively to average strain rate and standard deviation for major strain E_1_. Red curves and orange areas correspond respectively to average strain rate and standard deviation for minor strain E_2_. Data were filtered using a forward-backward linear filter. Filter coefficients were obtained from a low-pass digital Butterworth filter (3rd order, cut off frequency of 2Hz) that was first applied on data.

Strain rates measured on the various areas of the fascia lata during movement are illustrated on Figure 10. As for the strains, the strain rate measurements are very low on the anterior and lateral fascia for the subject 2019_285. Considering all the other tests, the maximum strain rates in flexion (positive values) and in extension (negative values) of the knee were close in absolute value, with an average difference of 1%/sec for E_1_ strain rate and 1.5% for E_2_ strain rate. Concerning the anterior and lateral fascia, these maximum strain rates can reach 6.3%/sec for E_1_ and 8.1%/sec for E_2_. As for the ITT, they can reach 10.5%/sec for E_1_ and 11.2%/sec for E_2_.

## Discussion

This *in situ* study is the first attempt to quantify the superficial strain field of fascia lata during passive leg movement. Motion analysis, stereovision and digital image correlation were combined to measure major, minor and maximum shear Green-Lagrange strains. These measurements were performed on three different locations of fascia lata: anterior, lateral and ITT.

The strain measurement by 3D surface digital image correlation in these conditions is challenging. Specific care was taken to get good quality images. A large depth of field was needed to limit the unfocussed area on the view of the anterior fascia area, as this area could have a pronounced curved shape. Lighting and exposure time were optimized to avoid, as much as possible, shiny areas due to the water content in the tissue, and the fascia had to be kept hydrated. In-depth hydration could not be controlled, but to ensure good surface hydration of the fascia along the tests, a sheet of saline-solution soaked paper systematically covered the fascia between tests. This protocol was performed to avoid loss of fluid that may affect fascia mechanical properties such as its stiffness (Schleip et al., 2012).

White makeup, mainly made of talc, glycerin and water, was also used to facilitate digital image correlation. This specific water-based makeup was indeed chosen because of its assumed lower effect on tissue hydration, however, its osmotic effect on fascia tissue remains unknown to the best of the authors’ knowledge. Connective tissue water content may affect its mechanical properties, and thus strain measurements. For instance, Chimich et al. (1992) showed that the viscoelastic behavior of ligaments soaked in phosphate-buffered saline solution was altered; Basser et al. (1998) used the osmotic stress technique to evaluate the relationship between hydration and the tensile stress in the collagen network. Thornton et al. (2001) showed that ligaments creep behavior increased with increased tissue hydration; and Masic et al. (2015) looked at changes in tensile forces resulting from osmotic pressure in tendon collagen. In the present study, we can consider that the water content within the fascia before application of the white mask was close to the natural one since cadaveric subjects were not frozen and only stored at 4°C before body preparation. However, water content is difficult to assess *in situ* on the whole specimens’ leg after application of makeup. Therefore, the osmotic effect of this white makeup on fascia water content and mechanical properties should be further investigated through tests on isolated samples, following an experimental protocol as proposed by Hammer et al. (2014) with the use of osmotic stress technique to assess the water content in ITT samples.

Moreover, another difficulty was to apply the knee flexion-extension without compromising the movement analysis or the strain field analysis on the fascia. This movement was applied manually by moving the ankle to avoid any constraint due to attachment of the femur or the lower leg but did not prevent from some variation of the angle between the pelvis and the femur. As the fascia lata involves both hip and knee joints, this hip angle variation could lead to the generation of strain noise that may contribute to the lack of reproducibility of mean strain during movement. To improve the set-up and increase reproducibility, a controlled device could be developed in order to guide the movement of the knee. With such system, it would then be possible to do the analysis without the optical motion capture system; only synchronization between stereovision and the new device would be needed. The challenge would be to have a device adaptable to different subjects’ anthropometries and that will not affect knee kinematics. Nevertheless, despite the manual application of movement, knee angular velocity was relatively reproducible, and representative of the movement surgeons could perform to test knee stability during surgery.

Cadaveric subjects were used in this study and hence the effect of postmortem storage on tissue mechanical properties may affect the presented results. The fascia lata is composed of highly oriented collagen fibers in different layers connected to each other with an extracellular matrix composed of proteoglycans (Pancheri et al., 2014). Both components may have deteriorated with death. Tuttle et al. (2014) investigated the effect of postmortem time on rabbit skeletal muscle mechanical and biochemical properties. As for collagen content, results indicate that it remained unaffected over 7 days postmortem. Another study by Nishimura et al. (1996) looked at the content of proteoglycans sheathing collagen fibrils and fibers. They showed that for beef stored at 4°C, proteoglycans content decreased by 40% within 7 days postmortem. In the present study, the maximum duration between time of death and time of test was 6 days, and during this period, subjects were kept at 4°C. Therefore, based on existing literature, we can hypothesize that for fascia lata the collagen content remained constant but that the links or lubricant between collagen fibers were degraded. This would probably imply that strain measurements are underestimated compared to what would be experienced *in vivo* on living tissue, since the viscosity properties provided by proteoglycans and that allow fibers to glide, progressively disappeared; but the impact on postmortem storage on fascia properties should be further investigated.

This study was performed on only three subjects. These subjects were geriatric people, presenting three different BMI and knee mobility ranges. At this stage of the study, inclusion criteria regarding for instance BMI, age or leg pathology were not considered, since the aim of the study was to get a first overview of the fascia strain field without preconditions. Nevertheless, the physical condition of these subjects prior to death could affect the results. Unfortunately, such information (regarding physical conditions or medical records as well as cause of death) of donors is unavailable, but we can still observe that two subjects had a low BMI suggesting muscular atrophy due to aging also known as sarcopenia, or due to a body weight loss related to cachexia. Such pathologies affect muscle tissue and its ability to contract (Muscaritoli et al., 2010; Tournadre et al., 2019). Moreover, age, gender and body weight have also been reported to affect collagen content within tissues and their mechanical properties. For patellar tendon, Couppé et al. (2009) showed that collagen content decreased with age while cross-linking increased, but that mechanical properties were maintained. For ligaments (Osakabe et al., 2001) and tendons (Guilbert et al., 2016), collagen was also found to decrease with age, but gender differences were also noticed with a higher decrease in the female group (Osakabe et al., 2001). Specifically regarding fascia, Zwirner et al. (2019) showed that for the iliotibial tract, tensile parameters did not depend on age, except for strain at failure in the male group; but other anthropometric parameters such as height should be considered when looking at fascia mechanical properties. Nonetheless, Wilke et al. (2019) used ultrasound measurements on young and older volunteers to show that fascia thickness changes with aging, depending on its location in the body and that fascia mechanical properties were affected. These changes are supposed to affect joint range of motion (decrease or enhance) but interactions between fascial thickness, age, body mass and flexibility should be further investigated. Therefore, even though physical condition of donors is unknown, fascia thickness or collagen content were not measured and the effect of age has been partially evaluated in the literature, we should acknowledge that this might have an influence on our results; especially for subject 2019_285 that had a higher BMI and a lower knee range of motion than the other two subjects, suggesting a stiffer fascia. However, regardless of these limitations and variability between subjects, interesting tendencies regarding measured strain levels and observed strain mechanisms can still be highlighted.

In comparison with the existing data in the literature, we can first consider the level of the fascia lata strains. The maximum value of 10 and 12% for the average major principal strain measured respectively on the lateral fascia and ITT are higher than the 9% reported by Gratz (1931; human iliotibial tract), the 9.7–10.3% reported by Hammer et al. (2012, 2014, 2016; human iliotibial tract) as tensile strain at failure, and the 8% reported by Eng et al. (2014; goat fascia lata) as damage tensile strain. It is still lower than the 15% reported by Zwirner et al. (2019; human iliotibial tract) as tensile strain at failure. However, it is smaller than the maximum tensile strain applied by Stecco et al. (2014) who performed tensile tests up to 30% on human crural fascia. It is also smaller than the 40.6% in the proximal-distal direction and 53.6% in the cranial-caudal direction reported by Henderson et al. (2015) on dog fascia lata, as tensile strain at failure. Butler et al. (1984) compared strain measurements techniques on fascia lata samples in tension, they reported 33% strain at failure for grip to grip measurement while it was only 14.5% when measured locally with an optical technique, which is closer to the values observed in this study even though not at failure. Within all the aforementioned results, high variability can be observed. One can wonder, on which part of the stress/strain curves obtained by *ex vivo* tensile testing, the strain states of the fascia lata measured *in situ*, are located. This also depends on the initial strain state of the samples tested *ex vivo*, probably also of fascia location in the body, preservation technique, species and fascia microstructure. These variations may also be related to strain rate and tissue viscosity. Tensile tests by Eng et al. (2014) and Pancheri et al. (2014) were performed at 0.15%/sec, those by Hammer et al. (2012, 2014, 2016) and Zwirner et al. (2019) at around 0.5%/sec while tests by Stecco et al. (2014) at 120%/sec. In the present study, strain rates were depending on knee angle, but average maximum values did not exceed 11.2%/sec, considering all tests and both E_1_ and E_2_. If we consider that the applied knee angular velocity was lower than physiological maximal velocity measured during gait, which can vary between 200 deg/sec to 400 deg/sec for healthy people, depending on gait speed (Mentiplay et al., 2018) the expected physiological strain rate of the fascia lata should be greater than the maximal values that we measured.

If we consider the shear strain mechanisms and sometimes the compressive strains larger than tensile strains, observed in this study, we can hypothesize that it is linked to the fascia microstructure. The fibers oriented in two different directions may be reticulated and act as local frames deformed during shear movements. This hypothesis lends itself less well to the explanation of the shear strain mechanism of the iliotibial tract. As it is made up of mostly collagen fibers in the longitudinal direction, we would have expected a major principal strain oriented in the longitudinal direction. But the observations from Otsuka et al. (2018) showing two main directions of fibers on the iliotibial tract (named lateral in Otsuka et al., 2018) seem in line with a shear mechanism explained by the fibers’ arrangement. This shear strain mechanism observed *in situ* may also explain why higher tensile strains have been measured in this study compared to the values measured by Eng et al. (2014) in the main fiber directions. Moreover, in most cases on anterior and lateral fascia lata, the minor strain measured on the fascia lata is larger than the major one in absolute value. This behavior has already been observed in an isolated sample of a hepatic capsule submitted to uniaxial tension (Jayyosi et al., 2014).

As previously presented, literature reports mainly tensile tests (Gratz, 1931; Butler et al., 1984; Hammer et al., 2012, 2014, 2016; Steinke et al., 2012; Pancheri et al., 2014; Stecco et al., 2014; Henderson et al., 2015; Ruiz-Alejos et al., 2016; Otsuka et al., 2018; Zwirner et al., 2019) or biaxial tensile tests (Eng et al., 2014; Pancheri et al., 2014) performed on fascia isolated samples while here, tensile but also shear strain mechanisms, especially on ITT, were highlighted during passive knee movement. Further studies on isolated samples, including bias tests or picture frame tests would allow the analysis of the in-plane shear mechanisms of fascia lata as it is usually done for textile composites (Boisse et al., 2017; Wang et al., 2020). Moreover, as it has been already performed on various fibrous connective membranes (Jayyosi et al., 2016; Lynch et al., 2017; Krasny et al., 2018), the detailed analysis of fascia lata fiber kinematics during such loading would also help in understanding the fiber links and tissue mechanical response. Different strain rates may also be applied to analyze the role of the microstructure in viscous phenomena.

The choice of the reference strain state is of great importance for the data analysis as it conditions the strain levels reached during the knee flexion-extension. But in many cases presented in this study, various strain mechanisms have been observed for two phases of the knee movement delimited by the reference posture of the knee (zero-gravity posture of the knee), which is used as the zero-strain state of fascia lata. This reference position of the knee was chosen as the same for the three subjects, but it may be subjected to inter-subject variability. Further analysis of the strain mechanisms obtained using various reference strain states could help in defining an optimum reference strain state of the fascia lata for each subject and then to identify maximum strain levels during knee movement in a more personalized manner. It is therefore difficult at present to decide on a position of the knee and hip that will result in a state of minimum strain of the fascia. Further *in situ* studies with more subjects would help to check if the changes in strain mechanisms correspond to a reference posture of the leg regarding the fascia lata strain-state.

For future *in situ* studies, inclusion criteria such as age, gender or body mass index could also be considered to perform an in-depth analysis of strain field based on the proposed methodology. Groups based on knee range of motion, or varus-valgus could also be studied, and the focus on a specific leg pathology or surgical practice could be considered. For instance, a surgical technique, at the knee level, that could benefit this work is the “pie-crusting” technique (Clarke et al., 2005; Nikolopoulos et al., 2015). Patients with varus or valgus knee may need this technique that consists of tissue release, and that can be done on ITT. Tissue release is done by inducing damage in the tissue, usually by making holes in it with a scalpel. The clinical question behind this technique is when to stop releasing the tissue. Thanks to our strain maps and optical motion capture system, strain release could be assessed as well as corresponding knee mobility and varus-valgus angle. Correlations observed experimentally could then be evaluated on patients during surgery.

A further example can be taken from hip surgery; the fascia is usually cut to get access to the joint. It is known that fascia lata participates in hip stability (Eng et al., 2014), therefore thanks to our proposed methodology and analysis, knowing the strain map of fascia around the hip may help in defining a hip position for minimal fascia strain, which could guide the surgeon for positioning patients in order to close fascia lata with minimal tension at the end of the procedure. For a defined hip position, we could evaluate the effect of incision on fascia strain release. Thanks to the obtained strain maps, insights on how hip position can affect strain release could be gathered, experiments could also be coupled with a numerical model of the leg that could reproduce fascia pre-strain on muscular tissue and reproduce both passive and active leg movement under physiological loadings, this last aspect will be challenging and require other experiments. To perform such tests related to surgical technique, possible issues regarding reproducibility in induced damage or incisions may occur. To limit this, these induced tears would need to be standardized using references to anatomical landmarks for the definition of scalpel incision location and size, and would need to be performed using the same technique carried out by the same operator for each subject. As done in the present study during knee flexion, the tracking of fascia lata strains before, during and after incisions using digital image correlation, would need to ensure an adequate field of view and good image quality along each step. Video recording could be done continuously, and knee movement performed before and after incision.

The methodology presented here is not an *in vivo* study; thus, both the contribution of the muscle activation and the effect of fat, superficial fascia and skin, on the deep fascia behavior are ignored. *In vivo* mechanical properties of fascia lata obtained using elastography have been reported (Otsuka et al., 2019) but such observations are still limited to a transversal section and small area of interest. Partial strain maps obtained *in situ* could be used as first validation data of passive numerical simulations.

To assess the effect of muscular activation on fascia strain field, which could then be implemented in leg numerical models, different approaches could be investigated. Firstly, using obtained strain maps *in situ*, specific areas of interest could be then studied *in vivo* using elastography measurements as proposed by Otsuka et al. (2019) and other electromyography measurements. We can also think of strain field acquisition on the skin, as proposed here on fascia. Various orientations should be studied, volunteers varying in gender, age groups and so on should be included, and medical imaging of their leg should be acquired in order to have an associated finite element model. Secondly, experiments following the same experimental protocol with the addition of muscular activation could be performed on animals. That kind of data is useful to develop and validate finite element models, but then the transition from animal data to application for human beings could be problematic due to difference in species, anatomy, or microstructures.

As a conclusion, this study presents some limitations but provides a step in understanding the strain mechanism of the fascia lata during knee movement. Further tests are planned to carry out the same analysis for various hip angles. The strain field measurement will also be applied to assess pre-strain and study strain release on different locations within fascia lata for clinical applications and for implementation in numerical models. More generally, the knowledge of the most released strain state of deep fascias could help surgeons when considering fascia closing after surgery or fascia release for surgical rehabilitations.

## Data Availability Statement

The datasets generated for this study are available on request to the corresponding author.

## Ethics Statement

Ethics approval was not required for this study since it is not required by French law. Mortal remains were obtained after the deceased gave their informed consent to donate them to science during their lifetime (law article R2213-13). The authors ensure that these experiments were performed with respect for these remains, and in proper health and safety conditions.

## Author Contributions

All authors contributed to the conception and design of the study, manuscript revision, read, and approved the submitted version. YS, AN, and KB-G performed the experiments and collected and treated raw data (digital image correlation and motion analysis). YS, AV, KB-G, and L-LG analyzed and discussed the data. YS, KB-G, and L-LG wrote the first draft of the manuscript.

## Conflict of Interest

The authors declare that the research was conducted in the absence of any commercial or financial relationships that could be construed as a potential conflict of interest.
