# Corrigendum: Strain Assessment of Deep Fascia of the Thigh During Leg Movement: An *in situ* Study

**DOI:** 10.3389/fbioe.2021.732609

**Published:** 2021-08-27

**Authors:** Yuliia Sednieva, Anthony Viste, Alexandre Naaim, Karine Bruyère-Garnier, Laure-Lise Gras

**Affiliations:** ^1^ Univ Lyon, Université Claude Bernard Lyon 1, Univ Gustave Eiffel, IFSTTAR, LBMC UMR_T9406, Lyon, France; ^2^ Hospices Civils de Lyon, Hôpital Lyon Sud, Chirurgie Orthopédique, Pierre-Bénite, France

**Keywords:** deep fascia of the thigh, digital image correlation, motion analysis, strain, in situ study, iliotibial tract, biomechanics

In the original article, there was a mistake in the legend for **Figure 9** as published. The positions of the knee relatively to reference position of 47° were inverted. An angle lower than 47° corresponds to an extended knee, and an angle greater than 47° corresponds to a flexed knee. The correct legend appears below.

In the original article, there was a mistake in **Figure 9** as published. Major principal strain E_1_ and minor principal strain E_2_ were inverted. The corrected **Figure 9** appears below.

Part of the abstract, results, and discussion sections have been revised according to the now correct results.

A correction has been made to **Abstract**:

“Fascia is a fibrous connective tissue present all over the body. At the lower limb level, the deep fascia that is overlying muscles of the outer thigh and sheathing them (fascia lata) is involved in various pathologies. However, the understanding and quantification of the mechanisms involved in these sheathing effects are still unclear. The aim of this study is to observe and quantify the strain field of the fascia lata, including the iliotibial tract (ITT), during a passive movement of the knee. Three fresh postmortem human subjects were studied. To measure hip and knee angles during knee flexion-extension, passive movements from 0° to around 120° were recorded with a motion analysis system and strain fields of the fascia were acquired using digital image correlation. Strains were computed for three areas of the fascia lata: anterior fascia, lateral fascia, and ITT. Mean principal strains showed different strain mechanisms depending on location on the fascia and knee angle. For the ITT, two strain mechanisms were observed depending on knee movement: compression is observed when the knee is extended relative to the reference position of 47°, however, tension and pure shear can be observed when the knee is flexed. For the anterior and lateral fascia, in most cases, minor strain is higher than major strain in absolute value, suggesting high tissue compression probably due to microstructural fiber rearrangements. This *in situ* study is the first attempt to quantify the superficial strain field of fascia lata during passive leg movement. The study presents some limitations but provides a step in understanding strain mechanism of the fascia lata during passive knee movement.”

A correction has been made to **Results, Strain Analysis, Strain Mechanisms, paragraph number 2**:

“On the anterior fascia (Figure 9A), for subject 2019_285, for which strain levels are lower, a tension mechanism is observed with a maximum average tensile strain around 1.5% and a lower absolute value of compressive strain around 0.5%. For subjects 2019_300 and 2019_308, absolute value of the compressive strain is greater than the average tensile strain when the knee is more flexed relative to the reference knee angle (red curves, knee angle > reference angle).”

**FIGURE 9:**
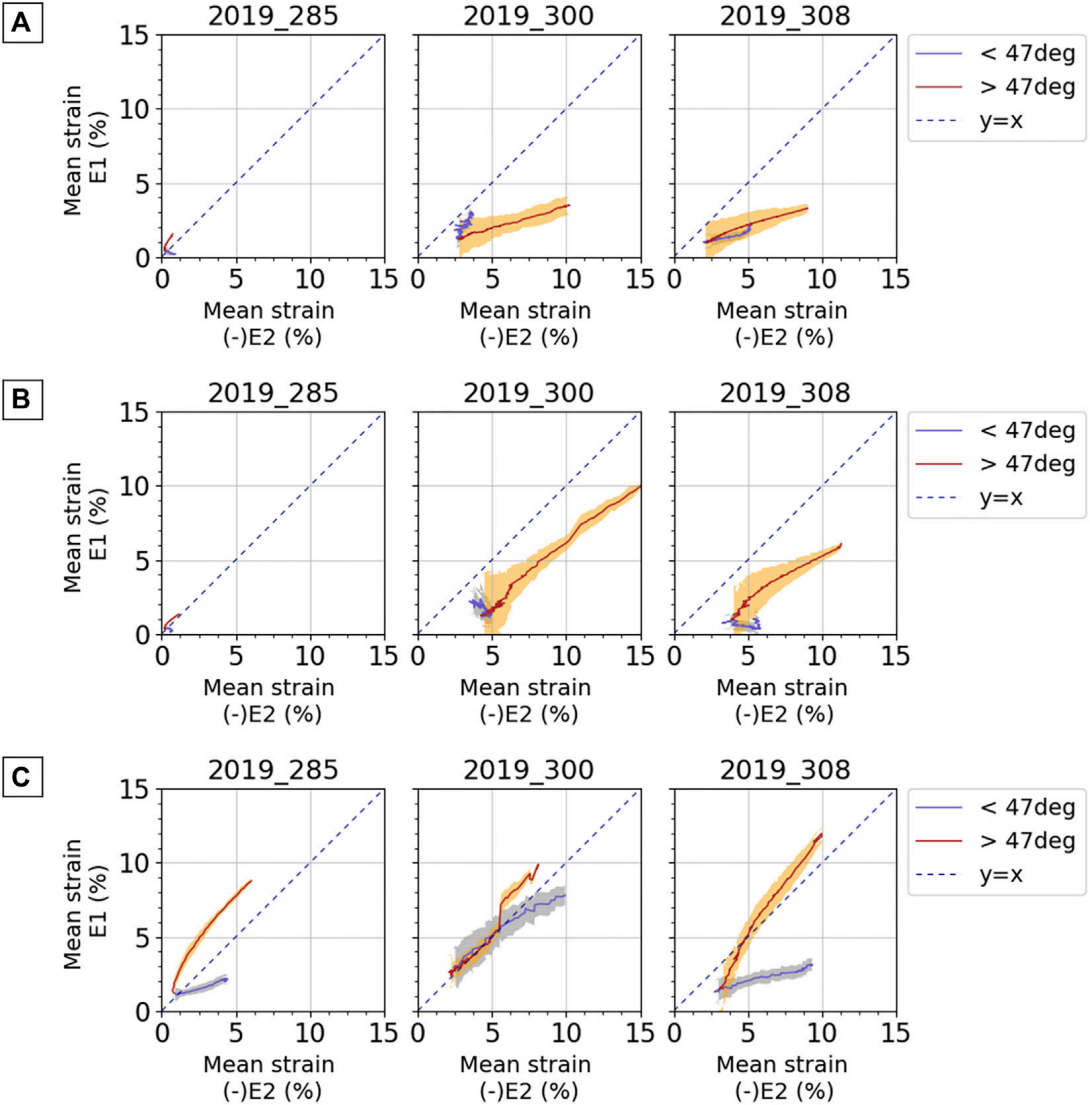
Average curves for mean major principal strain E_1_ against negative mean minor principal strain E_2_ for **(A)** anterior fascia, **(B)** lateral fascia, and **(C)** ITT. Results are presented for each subject and for each test. Blue curves and light blue areas correspond respectively to average values and standard deviation obtained for a knee angle lower than the reference position of 47°: extended knee. Red curves and orange areas correspond respectively to average values and standard deviation obtained for a knee angle greater than the reference position of 47°: flexed knee. Dashed line represents the y = x curve; when points are along this line, pure shear can be observed.

A correction has been made to **Results, Strain Analysis, Strain Mechanisms, paragraph number 3**:

“On the lateral fascia (Figure 9B), for the subjects 2019_300 and 2019_308, the same analysis as for the anterior fascia can be made, and compressive strain can reach up to 15% in absolute value. For the subject 2019_285, for which strain levels are lower, the strain mechanism of the fascia is pure shear with a ratio E_1_/(-E_2_) equal to 1 when the knee is more flexed relative to the reference knee angle (red curves, knee angle > reference angle).”

A correction has been made to **Results, Strain Analysis, Strain Mechanisms, paragraph number 4**:

“On the ITT (Figure 9C), the two phases of knee movement show different strain mechanisms for subjects 2019_285 and 2019_308. When the knee is more flexed relative to the reference knee angle (red curves, knee angle > reference angle), even if a tensile strain larger than the compressive strain may be observed, we can observe pure shear with a curve gradient close to 1. For subject 2019_300, the two phases of movement do not differentiate between strain mechanisms so clearly, but we can also observe some pure shear. Considering all tests, strain measurements on ITT show maximum tensile strain between 2 and 15% and compressive strain between 4 and 13% in absolute values.”

A correction has been made to **Discussion**, beginning of **paragraph number 7:**

“In comparison with the existing data in the literature, we can first consider the level of the fascia lata strains. The maximum value of 10 and 12% for the average major principal strain measured respectively on the lateral fascia and ITT are higher than the 9% reported by Gratz (1931; human iliotibial tract), the 9.7–10.3% reported by Hammer et al. (2012, 2014, 2016; human iliotibial tract) as tensile strain at failure, and the 8% reported by Eng et al. (2014; goat fascia lata) as damage tensile strain. It is still lower than the 15% reported by Zwirner et al. (2019; human iliotibial tract) as tensile strain at failure.”

The following text starting with “However, it is smaller than the maximum tensile strain applied by Stecco et al. (2014)…” is correct.

A correction has been made to **Discussion, paragraph number 8**:

“If we consider the shear strain mechanisms and sometimes the compressive strains larger than tensile strains, observed in this study, we can hypothesize that it is linked to the fascia microstructure. The fibers oriented in two different directions may be reticulated and act as local frames deformed during shear movements. This hypothesis lends itself less well to the explanation of the shear strain mechanism of the iliotibial tract. As it is made up of mostly collagen fibers in the longitudinal direction, we would have expected a major principal strain oriented in the longitudinal direction. But the observations from Otsuka et al. (2018) showing two main directions of fibers on the iliotibial tract (named lateral in Otsuka et al., 2018) seem in line with a shear mechanism explained by the fibers’ arrangement. This shear strain mechanism observed *in situ* may also explain why higher tensile strains have been measured in this study compared to the values measured by Eng et al. (2014) in the main fiber directions. Moreover, in most cases on anterior and lateral fascia lata, the minor strain measured on the fascia lata is larger than the major one in absolute value. This behavior has already been observed in an isolated sample of a hepatic capsule submitted to uniaxial tension (Jayyosi et al., 2014).”

The authors apologize for these errors and state that they do not change the scientific conclusions of the article in any way. The original article has been updated.

